# Carriage of multi-drug resistant bacteria among foreigners seeking medical care

**DOI:** 10.1038/s41598-018-27908-x

**Published:** 2018-06-21

**Authors:** Shmuel Benenson, Ran Nir-Paz, Mordechai Golomb, Carmela Schwartz, Sharon Amit, Allon E. Moses, Matan J. Cohen

**Affiliations:** 1 Department of Clinical Microbiology and Infectious Diseases, Hadassah-Hebrew University Medical Center, Jerusalem, Israel; 2 Heart Center, Hadassah-Hebrew University Medical Center, Jerusalem, Israel; 3 Clalit Health Services, Jerusalem district, Jerusalem, Israel

## Abstract

Medical tourism has a potential of spreading multi-drug resistant bacteria (MDR). The Hadassah Medical Center serves as a referral center for global medical tourists and for Palestinian Authority residents. In order to assess whether patients of these groups are more likely to harbor MDR bacteria than local residents, we reviewed data from all patients admitted to our institution between 2009 and 2014. We compared MDR rates between countries of residency, controlling for gender, age, previous hospitalization and time from admission to MDR detection. Overall, among 111,577 patients with at least one microbiological specimen taken during hospitalization, there were 3,985 (3.5%) patients with at least one MDR-positive culture. Compared to Israeli patients, tourists and patients from the Palestinian Authority had increased rates of MDR positivity (OR, 95%CI): 2.3 (1.6 to 2.3) and 8.0 (6.3 to 10.1), respectively. Our data show that foreign patients seeking advanced medical care are more likely to carry MDR bacteria than the resident population. Strategies to minimize MDR spread, such as pre-admission screening or pre-emptive isolation should be considered in this population.

## Introduction

Multi-drug resistant (MDR) organisms are a global threat^1,2^, leading to prolonged hospital stay, treatment failure, excess in-hospital death, and increased economic costs^3–5^. In order to prevent MDR bacteria cross-transmission and spread, hospitals employ isolation policies, including identification and preventive isolation measures of patients at high risk of carrying these bacteria^6^.

Medical tourism is not new; however, as a result of the shrinking global village, far more people seeking expert medical care travel between countries. Thus, there is an increased risk that medical tourism could lead to geographically dispersed outbreaks of communicable diseases, including the spread of MDR bacteria to non-endemic facilities^7,8^. Multi-drug-resistant Gram-negative bacteria, including MBL-producing *Pseudomonas aeruginosa*, extended-spectrum beta lactamase and carbapenemase-producing Enterobacteriaceae and multi-drug resistant *Acinetobacter baumannii*, have been circulating the globe in recent years^9–13^.

Traditional risk factors for carriage of MDR bacteria include previous carriage, recent hospital stay, recent exposure to antibiotics and long-term care facility residence^14^. Currently, medical tourists are not considered a separate group that might require pre-emptive contact isolation^15^. Until now, most of the evidence regarding the potential threat of spreading resistant bacteria was based on single source epidemics associated with one traceable pathogen, or a resistance mechanism which was newly introduced to a country.

Hadassah-Hebrew University Medical Center serves as a referral center for global medical tourists and for residents of the Palestinian Authority. We sought to assess whether these patient populations are more likely to harbor resistant bacteria than local patients and thus may necessitate pre-emptive isolation until carriage status is determined.

## Methods

### Setting and patients

The Hadassah-Hebrew University Medical Center consists of two academic hospitals with a total of 1100 inpatient beds. Ein-Kerem campus is a 750 inpatient bed tertiary care center and Mount-Scopus campus is a 350 inpatient bed community hospital. The hospitals provide adult and pediatric medical, surgical and psychiatric services, including medical and surgical subspecialties, oncology, hematology, neurosurgery, transplantations and comprehensive outpatient and ambulatory services.

**Figure 1:**
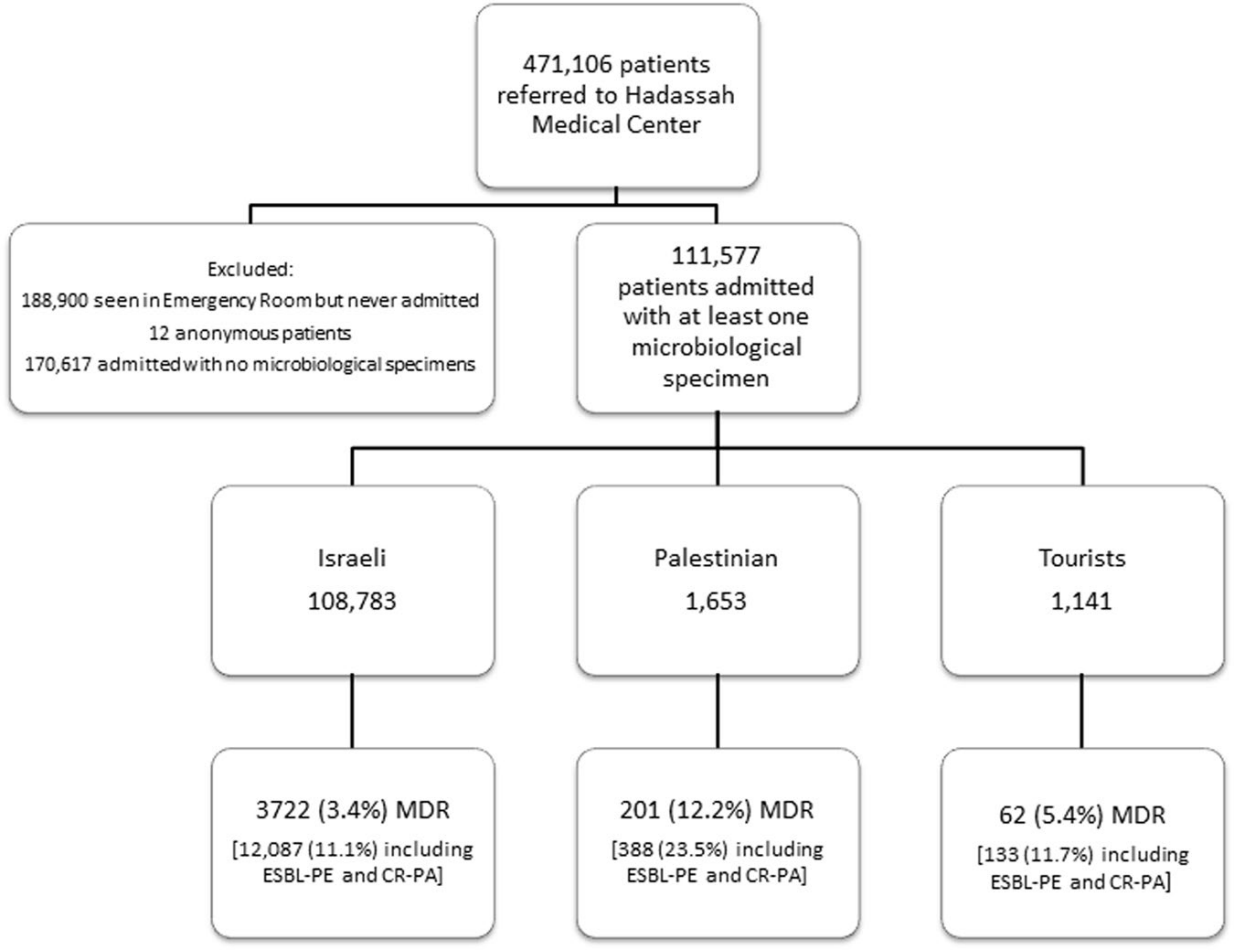
Flowchart of patient selection, exclusion and multi drug resistant (MDR) rates. The study period extended from July 1^st^ 2009 to December 31^st^ 2014. Only patients admitted at least once and from whom at least one microbiological specimen was obtained were included in the study. Patients were classified into three groups and in each, the rate of MDR bacteria was calculated twice, once excluding and once including carbapenem-resistant *Pseudomonas aeruginosa* (CR-PA) and extended-spectrum-beta-lactamase producing Enterobacteriaceae (ESBL-PE) (see text).

The source population from which we derived the study population included all patients who arrived at both centers of the Hadassah-Hebrew University Medical Center between January 1^st^ 2009 and December 31^st^ 2014, including the emergency department, regular hospitalization and daycare. In order to account for a six-month hospitalization period prior to the inclusion date, the study period was limited to July 1^st^ 2009 through December 31^st^ 2014. The analysis was performed only on patients who were admitted as inpatients at least once during the study period and from whom at least one microbiological specimen was obtained during this time (Fig. 1). All data in the study was collected from the computerized institutional repository. We extracted age, gender and nationality as well as hospital-referrals and intensive care unit (ICU) stays during the study period, and the prior six months.

Patient nationality was defined as Israeli citizens, residents of the Palestinian Authority and foreign tourists. Patients from the Palestinian Authority usually receive care from Palestinian medical services and arrive at Israeli medical centers for more advanced investigations and treatments. In all cases of foreign tourists found to be MDR positive, we examined the patient files to assess whether they were *medical* tourists who came to Israel for treatment. Patients were stratified into five age-groups: 0–18, 19–40, 41–65, 66–80 and above 80 years of age.

The Hadassah Hebrew University Medical Center ethics committee approved the study and waived the requirement for informed consent.

### Specimens and multi drug resistance (MDR)

Microbiological data from all clinical and screening specimens was extracted from the microbiology laboratory computerized database (WHONET)^16^. Clinical specimens are obtained based on patient’s clinical status and according to the discretion of the attending physician. Screening is carried out upon admission for every patient with risk factors for MDR carriage (hospital admission in previous six months or residency in long-term-care facility). These risk factors are assessed by the nursing staff upon admission and define the necessity for screening. MDR Screening is also performed for all intensive care unit patients upon admission and once weekly. The data included all specimens sent for culture and *Clostridium difficile* toxin (CDT) tests. In our hospital, bacteria considered multi drug resistant (MDR) that require contact isolation precautions include: carbapenem-resistant Enterobacteriaceae (CRE); carbapenem-resistant *Acinetobacter baumannii* (CR-ACIN); methicillin-resistant *Staphylococcus aureus* (MRSA); vancomycin-resistant Enterococci (VRE) and *Clostridium difficile*. For simplicity, in this report these five bacteria groups are all termed MDR. Additionally, we present secondary data regarding carbapenem-resistant *Pseudomonas aeruginosa* (CR-PA) and extended-spectrum-beta-lactamase producing Enterobacteriaceae (ESBL-PE), which in our institution do not necessitate contact isolation and are not defined as MDR bacteria in this study’s primary analysis.

### Microbiological methods

Resistant bacteria isolates reported in this study were of two origins: a. Strains from clinical samples with resistance to the antibiotic of interest (i.e. carbapenems for CRE and *A. baumannii*, cefoxitin and oxacillin for MRSA, vancomycin for VRE, and 3^rd^ generation cephalosporins for ESBL-PE). b. Strains with the above mentioned resistance identified as part of the institutional rectal/nasal screening protocols

The clinical samples were processed according to the routine laboratory diagnostic flow, which included identification by classical biochemical methods relevant at that time^17^ or matrix-assisted laser desorption/ionization time-of-flight (MALDI-TOF). Antimicrobial sensitivity testing was performed using the disk-diffusion method according to CLSI standards in the concordant years^18,19^. Suspected CRE were further verified for carbapenemase production using the modified Hodge test^11,20^. The screening samples were processed as follows: rectal/nasal swabs were plated onto selective chromogenic media (ChromAgar KPC/ChromAgar ESBL/ChromAgar MRSA, HyLabs, Rehovot, Israel) and incubated at 35 °C for 18–24 hours. Suspected colonies were identified using classical biochemical methods relevant at that time or MALDI-TOF^17^. Following identification of CRE, *A. baumannii* and ESBL-PE, a designated antimicrobial sensitivity testing panel was used to verify resistance to carbapenems and 3^rd^ generation cephalosporins, respectively. Additionally, for CRE, a modified Hodge test was performed to verify the production of carbapenemases^20^. For VRE, rectal swabs were plated on VRE media (Novamed, Jerusalem, Israel) and incubated at 35 °C for 18–24 hours. Esculin hydrolyzing colonies were further identified by MALDI-TOF to exclude enterococci other than *E. faecium* and *E. fecalis*. Prior to 2012, CDT was diagnosed using an enzyme immuno-assay (Meridien Life Sciences, TN, USA). From 2012 onwards, CDT was diagnosed using a home-brewed Sybr green based real-time PCR performed on fecal DNA extracted by easyMag (bioMerieux, NC, USA).

### Data Analysis

The demographic data repository and the clinical microbiology lab repository were cross-referenced. Initially we recorded for every patient whether any culture specimen or CDT test was performed. Among patients with at least one specimen tested, we identified MDR positive specimens and recorded the date of the first positive specimen. Patients with at least one positive MDR isolate were defined MDR-positive patients. The date of the first MDR bacterium detected was defined as the patient’s index date.

In order to assess community acquisition vs. hospital acquisition, the time from hospital admission to the index date was recorded for MDR-positive patients. The dates were stratified into three time-groups: up-to 96 hours from admission, 96 hours to one week or greater than one week. We expanded the commonly used time span of 72 hours to 96 hours, taking into account a possible delay between the actual time of culturing and registration of the specimen in the lab.

We calculated the rate of MDR-positive patients (out of all included patients) per population, age and time groups. In addition, we calculated the rate of the first positive specimen for each specific MDR pathogen per patient and present these rates per nationality. Since more than one MDR bacteria can be present in a single patient, the sum of the rates per specific MDR pathogen exceeds the crude rate of MDR-positive patients.

We attempted to account for exposure to a healthcare facility prior to MDR bacteria identification. To this end, we counted the total number of admissions to our hospitals in the six months preceding the first MDR isolation. Hospitalization data was available for prior admissions only to our medical center. In order to assess the role of prior healthcare exposure on the risk of MDR positivity, a comparison with MDR-negative patients was required. As there is no index date for MDR-negative patients, in order to minimize possible biases, we defined the earliest admission date with the maximal number of prior six-month hospitalizations as the index date of a given patient.

In addition, we examined the rate of CDT positivity among all patients for whom the test was performed.

Logistic regression was performed to control for age and gender and to examine if nationality is associated with MDR bacteria carriage risk. When analyzing this model for all MDR bacteria, we also controlled for previous hospital exposure. However, when analyzing this model for a specific MDR pathogen, previous hospital exposure was excluded because some patients acquired a specific pathogen long after acquiring a previous pathogen and therefore previous admissions were not relevant to the latter acquisition. In each model we generated a random sample of controls three times larger than the case group, in order to prevent data skewing and mathematically biased results.

### Data availability

The datasets generated during and/or analyzed during the current study are available from the corresponding author on reasonable request.

## Results

During the study period, there were 942,702 referrals of 471,106 patients to the Hadassah-Hebrew University Medical Center; among them 111,577 patients met the inclusion criteria (Fig. 1).

Patient characteristics are presented in Table 1. Most patients were not admitted to our institution in the prior six months; however, previous admissions were more common among Israeli patients. Palestinian Authority patients had a higher proportion of younger patients while Israelis included more patients above 80 years of age. ICU stay in the prior six months was more often found among Palestinian patients.

**Table 1:** Patient characteristics (N = 111,577).

Variable	N (%)			p-value
Residence	Israeli	Palestinian Authority	Foreign tourists	
Number of patients	108,783	1653	1141	
Female gender	61,754 (57%)	641 (39%)	572 (50%)	<0.001
Age group (years)				
Up to 18	31,488 (29)	653 (40)	162 (14)	<0.001
18 to 40	31,115 (29)	465 (28)	291 (26)	
40 to 65	21,974 (20)	401 (24)	389 (34)	
65 to 80	14,840 (14)	108 (7)	227 (20)	
above 80	9,366 (9)	26 (2)	72 (6)	
Previous 6 months admissions				
None	61,872 (57)	1,152 (70)	867 (76)	<0.001
One	31,839 (29)	267 (16.2)	176 (15)	
More than one	15,072 (14)	234 (14)	98 (9)	
ICU stay, previous 6 months	4,661 (4.3)	107 (6.5)	37 (3.2)	<0.001

ICU, intensive care unit.

Overall, there were 3,985 (3·5%) patients with MDR bacteria. MDR positivity rates were highest among Palestinians (12·2%) followed by tourists (5·4%) and Israeli residents (3·4%) (p < 0.001) (Fig. 1). Including ESBL-PE and CR-PA there were 12,608 (11·3%) MDR positive patients: 10,271 ESBL-PE and 826 CR-PA.

The distribution of MDR positivity (MDR positive patients out of all included patients) per stratum of time from hospital admission to first MDR incidence is presented in Table 2. In 1,722/3,985 (43·2%) MDR events, identification occurred within 96 hours from hospital admission. In the additional analysis including ESBL-PE and CR-PA, 7,646/12,608 (60·6%) of the events were identified within 96 hours from hospital admission.

**Table 2:** Multi drug resistant carriage rates.

Variable	N (%)				p-value
Residence	Israeli	Palestinian Authority	Foreign tourists	Total	
Number of patients	108,783	1,653	1,141	111,577	
MDR positivity (overall)	3,722 (3.4)	201 (12.2)	62 (5.4)	3,985 (3.6)	<0.001
MDR positivity according to time after hospital admission					
Up to 96 hours	1,590 (1.5)	96 (5.8)	36 (3.2)	1,722 (1.5)	<0.001
96 hours–1 week	352 (0.3)	16 (1.0)	4 (0.4)	372 (0.3)	
More than one week	1,780 (1.6)	89 (5.4)	22 (1.9)	1,891 (1.7)	
MDR-specific positivity rates					
CRE	769 (0.7)	31 (1.9)	19 (1.7)	819 (0.7)	<0.001
VRE	616 (0.6)	25 (1.5)	16 (1.4)	657 (0.6)	<0.001
MRSA	1,205 (1.1)	58 (3.5)	13 (1.1)	1,276 (1.1)	<0.001
CR_ACIN	770 (0.7)	73 (4.4)	17 (1.5)	860 (0.8)	<0.001
C. difficile*	1,393 (1.3)	56 (3.4)	16 (1.4)	1,465 (1.3)	<0.001

*C. difficile positivity rates among patients whose stool samples were tested: Israeli 15.7%, Palestinian 17.6%, foreign tourists 9.8%.

MDR, multi-drug resistant; CRE, carbapenemase-producing Enterobacteriaceae; VRE, vancomycin-resistant Enterococci; MRSA, methicillin-resistant *Staphylococcus aureus*; CR-ACIN, carbapenem-resistant *Acinetobacter baumannii*.

*C. difficile* toxin tests were performed for 9339 (8·4%) of all included patients. *C. difficile* was the most prevalent MDR bacterium (1·3%) followed by MRSA (1·1%) (Table 2). Per specific MDR bacteria, Palestinian patients had the highest carriage rates for all bacteria, and CR-ACIN was the most prevalent (4·4%). For most MDR positive patients, only a single MDR type was identified (3160, 79·3%). Bacteremia was the first presentation of MDR infection in 260/3,985 (6·5%) cases.

Distribution of MDR positive foreign tourists according to geographic origin was as follows: East Europe, 46/586 (7·8%); North America and Western Europe, 12/369 (3·2%); all other, 4/179 (2·2%).

Among MDR positive foreign patients 49/62 (79%) were medical tourists. Among medical tourists, MDR were identified on their first visit to our institution in 28/49 (57%) cases. Among non-medical tourists MDR was identified during their first visit to our institution in only 1/13 (8%) cases.

**Figure 2:**
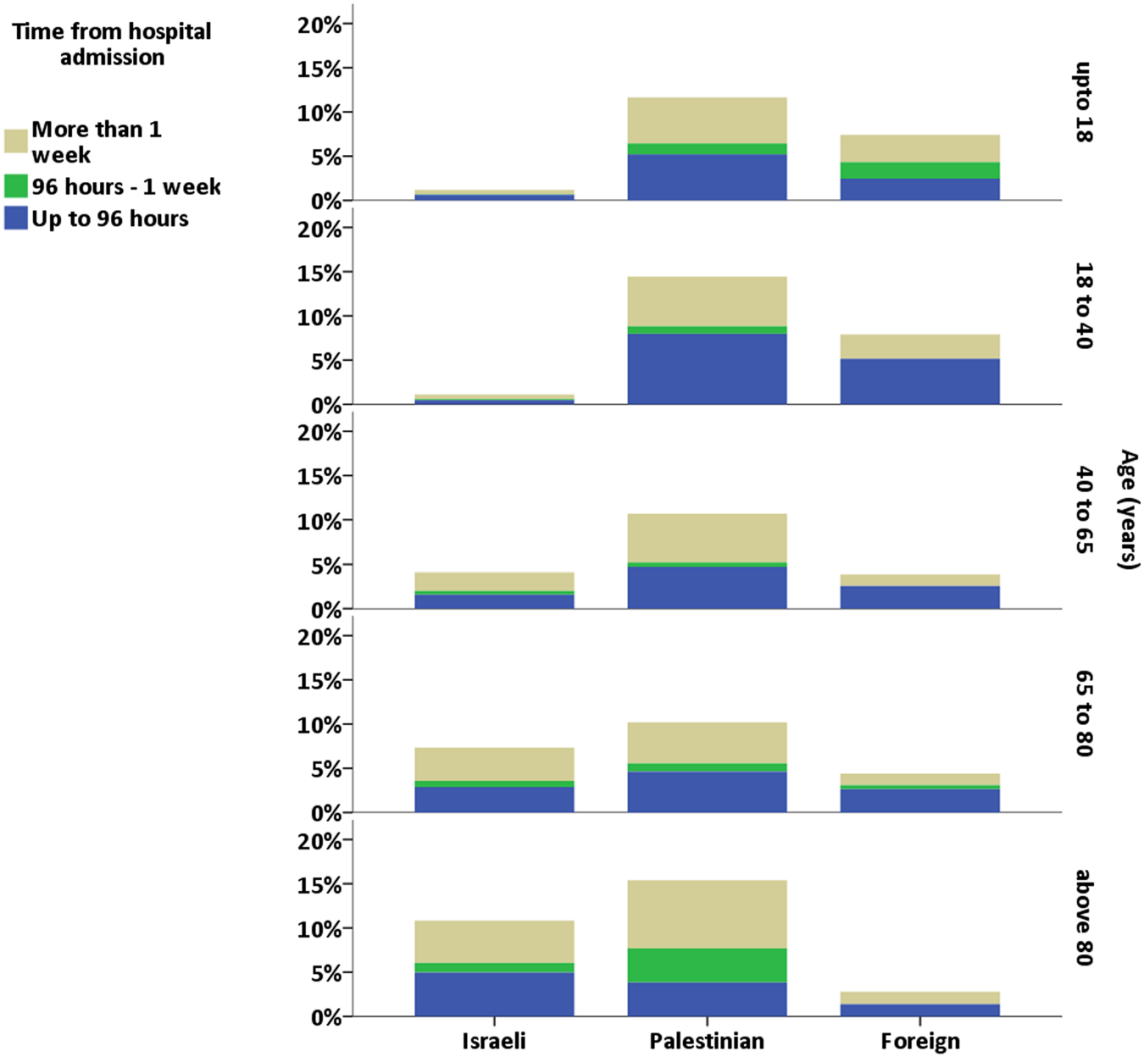
Multi drug resistant (MDR) prevalence per age group and source population. Each panel depicts MDR prevalence per age group. Columns depict the cumulative MDR rate in each age group, per patient nationality. The color specifications depict the time from hospital admission to first identification of MDR bacteria. Palestinian patients were found to have the highest rates of MDR bacteria in all age groups. Among patients up to 40 years old, foreign tourists had higher MDR rates than Israeli patients. In the older age groups, MDR rates were lower among foreign tourists. In most age groups about half of MDR bacteria were identified in the first 96 hours after admission.

Positivity rates, stratified by nationality, age group and time from admission, are presented in Fig. 2 (data shown in Supplementary Table S1). MDR positivity was highest among Palestinians in all age groups. MDR positivity among Israelis increased with age. In contrast, among foreign patients, MDR positivity rates were highest in the younger patients and rates decreased with age. In Supplementary Figure S1 we present similar results with inclusion of ESBL-PE and CR-PA.

The results of the regression model are presented in Table 3. Controlling for gender, age and previous hospital admission (only in the model for any MDR bacteria), Palestinian and foreign tourists were associated with increased risk of MDR positivity (OR, 95%CI: 8·0, 6·3 to 10·1; 2·3, 1·6 to 3·3, respectively). Results were similar when limiting the analysis to patients among whom MDR were identified during the first 96 hours after admission (data not shown) and for a specific bacteria. More than one previous hospitalization in the prior six months was also associated with increased rates of MDR positivity (OR 1·6, 95%CI 1·4 to 1·8).

**Table 3:** Regression models assessing the risk of multi-drug resistant bacteria positivity.

Variable	Odds Ratio (95%CI)					
Bacteria	Any MDR bacteria*	CRE	VRE	MRSA	CR-ACIN	*C. difficile*
Female	0.68 (0.63 to 0.74)	0.65 (0.54 to 0.78)	0.79 (0.65 to 0.96)	0.6 (0.52 to 0.68)	0.48 (0.41 to 0.56)	0.8 (0.7 to 0.92)
Residence (reference: Israeli patients)						
Palestinian	8.0 (6.3 to 10.1)	6.8 (3.8 to 12.1)	7.3 (3.8 to 13.7)	5.1 (3.4 to 7.6)	13.5 (8.4 to 21.7)	5.7 (3.8 to 8.5)
Foreign tourists	2.3 (1.6 to 3.3)	4.9 (2.4 to 10.1)	2.5 (1.2 to 5.1)	0.85 (0.45 to 1.65)	2.7 (1.4 to 5.4)	1.1 (0.7 to 1.9)
Age (reference: age up to 18)						
18 to 40	1.05 (0.9 to 1.2)	1.9 (1.2 to 2.8)	2.3 (1.5 to 3.6)	0.8 (0.63 to 1.0)	3.5 (2.5 to 5.1)	0.9 (0.7 to 1.1)
40 to 65	3.2 (2.8 to 3.6)	7.6 (5.3 to 11.1)	10.9 (7.2 to 16.4)	2.1 (1.7 to 2.6)	9.5 (6.7 to 13.3)	3 (2.4 to 3.8)
65 to 80	5.9 (5.2 to 6.7)	19 (13.1 to 27.4)	16.6 (11 to 25.2)	4.0 (3.2 to 4.9)	11.6 (8.3 to 16.4)	6.4 (5.2 to 8)
above 80	9.1 (7.9 to 10.4)	26 (17.8 to 38.1)	18.2 (11.8 to 28.2)	4.6 (3.7 to 5.8)	15.8 (11 to 22.8)	11.5 (9.3 to 14.5)

MDR, multi-drug resistant; CRE, carbapenem resistant Enterobacteriaceae; VRE, vancomycin resistant Enterococci; CR-ACIN, carbapenem resistant *Acinetobacter baumannii*.

*The model was also controlled for hospitalization in the prior six months.

## Discussion

In this study, which summarizes data from six years, including almost a million hospital referrals from nearly half a million patients, we found that patients of foreign origin, including residents of the Palestinian Authority and foreign tourists, have higher rates of MDR bacteria than Israeli patients. The chances of carrying MDR were higher among Palestinian Authority patients than patients arriving from other countries. These data are in accord with earlier reports of high MDR prevalence in the Palestinian Authority^21,22^.

Though non-Israeli patients had less prior exposure to our institution, we believe this does not reflect their true exposure to medical institutions prior to arrival to our center. Among foreign tourists, most MDR positivity was found among *medical* tourists, with the younger age groups presenting higher rates of MDR-bacteria. Most medical tourists with MDR bacteria were from East Europe, where there have been reports of high rates of MDR bacteria^23^. We hypothesize that most Palestinians seeking medical care in our hospitals are a selected group who were exposed to other medical institutions prior to their admission. Therefore, we can probably relate to the majority of patients in this group as *medical* tourists, although data regarding previous hospitalizations of Palestinian patients in the Palestinian Authority or elsewhere was not available.

Medical tourists are patients seeking medical treatment out of their homeland who have been often exposed to medical care in institutions in their country of origin before their referral to other countries. As hospitals are reservoirs for MDR bacteria, medical tourists are a particular concern regarding the spread of MDR bacteria between countries^23^. In addition it has been shown that patients with a recent history of travel to MDR-endemic areas (but not admitted to healthcare facilities abroad) were found to have increased rates of carriage of MDR organisms. The highest prevalence was observed in travelers returning from southern Asia^2,7,9^.

We suggest that the higher MDR bacteria rates among foreign patients found in our study reflect increased risk due to frequent hospitalizations as well as high MDR bacteria prevalence in their places of origin. Introduction of resistant bacteria from Palestinians and tourists has been previously reported^24,25^.

In a cross-sectional survey of nasal *S. aureus* carriage in healthy children and their parents throughout the Gaza strip (which is part of the Palestinian Authority), MRSA was detected in 12% of the study population and 45% of *S. aureus* isolates were MRSA^22^. Antibiotic self-medication practices and over the counter purchase of antibiotics, reported as common in the Palestinian Authority, probably contribute to the high MDR prevalence^26,27^. Additionally, accessibility to broad spectrum antibiotics via direct donations may also attribute, and thus such observation can be implicated to patients arriving from other low resource countries.

Healthcare institutions should have sound infection prevention strategies to mitigate the risk of dissemination of MDR organisms from patients previously hospitalized in other countries. In order to prevent the spread of MDR bacteria, pre-emptive prevention policies should be established. One such option would require medical tourists to undergo carriage screening before arriving at the expert centers. This course would require standard and validated specimen collection, culturing and assessment methods. In many instances, medical tourists come from less privileged areas where there is less access (geographical or financial) to well standardized medical microbiological services. Therefore, a more practical approach would be to screen foreign patients upon admission, especially those from known areas endemic for MDR bacteria, and to apply contact precautions pending result of screening cultures^28^. This strategy is known to be applied in some countries with low MDR prevalence^3,29–31^.

This study has several limitations. Working with such a large data repository does not allow in depth case analysis. Therefore, information on cause of admission, comorbidities and prior antibiotic exposure was not included in this report. The denominator of the calculated carriage rates was the number of patients who were sampled and not all admitted patients; cultures were collected from 40% (111,577/282,194) of admitted patients (Fig. 1). Additionally, patients for whom clinical cultures were taken but were MDR negative cannot be ruled out as MDR bacteria carriers. However this (non-differential) bias exists in all study groups and therefore would only serve to underestimate the real differences between patient groups^32^. Indeed, we found high rates of MDR bacteria among non-Israeli patients also in the third time interval (more than one week after admission) probably representing an undetected MDR carriage state.

Among Palestinians, we do not have enough information to establish which patients were medical tourists and whether they were previously exposed to healthcare facilities in the Palestinian Authority. In either event, this patient group had markedly higher MDR bacteria rates. Identifying specific risk factors associated with MDR bacteria carriage (e.g. previous hospitalization, long term care facility residence, and previous antibiotic treatment) could improve targeted screening and isolation resources. Regional initiatives currently underway between Hadassah-Hebrew University Medical Center and several medical institutions in the Palestinian Authority are intended to improve our information in these areas and promote better infection control practices in all cooperating parties.

Data on prior antimicrobial exposure and prior hospital admission could have provided better identification of patient groups for focused screening of MDR carriage. However, definition and criteria of these characteristics are fluid, and in many cases, the available medical documentation, if any, is poorly understood. We think that our proposal to identify non-local patients as a group with higher MDR propensity is more practical for implementation in other countries.

In conclusion, we found that in addition to the general risk factors of MDR bacteria carriage, foreign patients seeking advanced medical care are more likely to carry MDR bacteria than residents. Institutions should identify their specific specialties that attract medical tourists, and perform a stratified risk analysis for screening, pre-emptive isolation and decolonization of specific patients, based on gender, age, previous hospitalization and country of origin. Maps of country-specific MDR prevalence should be publically available and routinely updated in order to provide better care for medical tourists and reduce the risk of bacterial transmission in the host countries.

## Electronic supplementary material

Supplementary information
