# Supplementary material for: Carriage of multi-drug resistant bacteria among foreigners seeking medical care
Source: Sci Rep. 2018 Jun 21;8:9471. doi: 10.1038/s41598-018-27908-x (PMC6013425; doi:10.1038/s41598-018-27908-x)
Supplement: Supplementary file 1 — Supplementary information [file 41598_2018_27908_MOESM1_ESM.pdf]

Carriage of multi-drug resistant bacteria among foreigners seeking medical care

Shmuel Benenson, MD, MSc, [1] Ran Nir-Paz, MD, [1] Mordechai Golomb, MD,[2] Carmela Schwartz, RN, MPH,[1] Sharon Amit, MD, PhD,[1] Allon E Moses, MD,[1] and Matan J Cohen, MD, PhD [1,3]

[1]Department of Clinical Microbiology and Infectious Diseases, [2]Heart Center, Hadassah-Hebrew University Medical Center, [3]Clalit Health Services, Jerusalem district, Jerusalem, Israel

Supplementary table and figure

Supplementary Table 1. Distribution of MDR bacteria positivity among study groups, stratified per age and time from hospital arrival

Age group (years)	Time from arrival	Israeli N (%)	East Jerusalem N (%)	Palestinian N (%)	Foreign N (%)	Total N (%)
Up to 18	MDR up to 96 hours	107 (0.5%)	94 (1%)	34 (5.2%)	4 (2.5%)	239 (0.7%)
	MDR 96 hours to week	9 (0%)	11 (0.1%)	8 (1.2%)	3 (1.9%)	31 (0.1%)
	MDR more than week	93 (0.4%)	58 (0.6%)	34 (5.2%)	5 (3.1%)	190 (0.6%)
	no MDR bacteria	21425 (99%)	9685 (98.3%)	577 (88.4%)	150 (92.6%)	31837 (98.6%)
18 to 40	MDR up to 96 hours	106 (0%)	41 (0%)	37 (10%)	15 (10%)	199 (0%)
	MDR 96 hours to week	26 (0%)	8 (0%)	4 (0%)	0 (0%)	38 (0%)
	MDR more than week	122 (0%)	42 (0%)	26 (10%)	8 (0%)	198 (0%)
	no MDR bacteria	24091 (100%)	6676 (100%)	397 (90%)	268 (90%)	31432 (100%)
40 to 65	MDR up to 96 hours	271 (0%)	77 (0%)	19 (0%)	10 (0%)	377 (0%)
	MDR 96 hours to week	74 (0%)	21 (0%)	2 (0%)	0 (0%)	97 (0%)
	MDR more than week	352 (0%)	106 (0%)	22 (10%)	5 (0%)	485 (0%)
	no MDR bacteria	16727 (100%)	4332 (100%)	359 (90%)	374 (100%)	21792 (100%)
65 to 80	MDR up to 96 hours	354 (0%)	75 (0%)	5 (0%)	6 (0%)	440 (0%)
	MDR 96 hours to week	83 (0%)	18 (0%)	1 (0%)	1 (0%)	103 (0%)
	MDR more than week	458 (0%)	101 (0%)	5 (0%)	3 (0%)	567 (0%)
	no MDR bacteria	11543 (90%)	2218 (90%)	97 (90%)	217 (100%)	14075 (90%)
above 80	MDR up to 96 hours	404 (0%)	61 (10%)	1 (0%)	1 (0%)	467 (0%)
	MDR 96 hours to week	92 (0%)	10 (0%)	1 (0%)	0 (0%)	103 (0%)
	MDR more than week	392 (0%)	56 (10%)	2 (10%)	1 (0%)	451 (0%)
	no MDR bacteria	7476 (90%)	888 (90%)	22 (80%)	70 (100%)	8456 (90%)
Total	MDR up to 96 hours	1242 (0%)	348 (0%)	96 (10%)	36 (0%)	1722 (0%)
	MDR 96 hours to week	284 (0%)	68 (0%)	16 (0%)	4 (0%)	372 (0%)
	MDR more than week	1417 (0%)	363 (0%)	89 (10%)	22 (0%)	1891 (0%)
	no MDR bacteria	81262 (100%)	23799 (100%)	1452 (90%)	1079 (90%)	107592 (100%)

MDR, multi-drug resistant

Supplementary figure

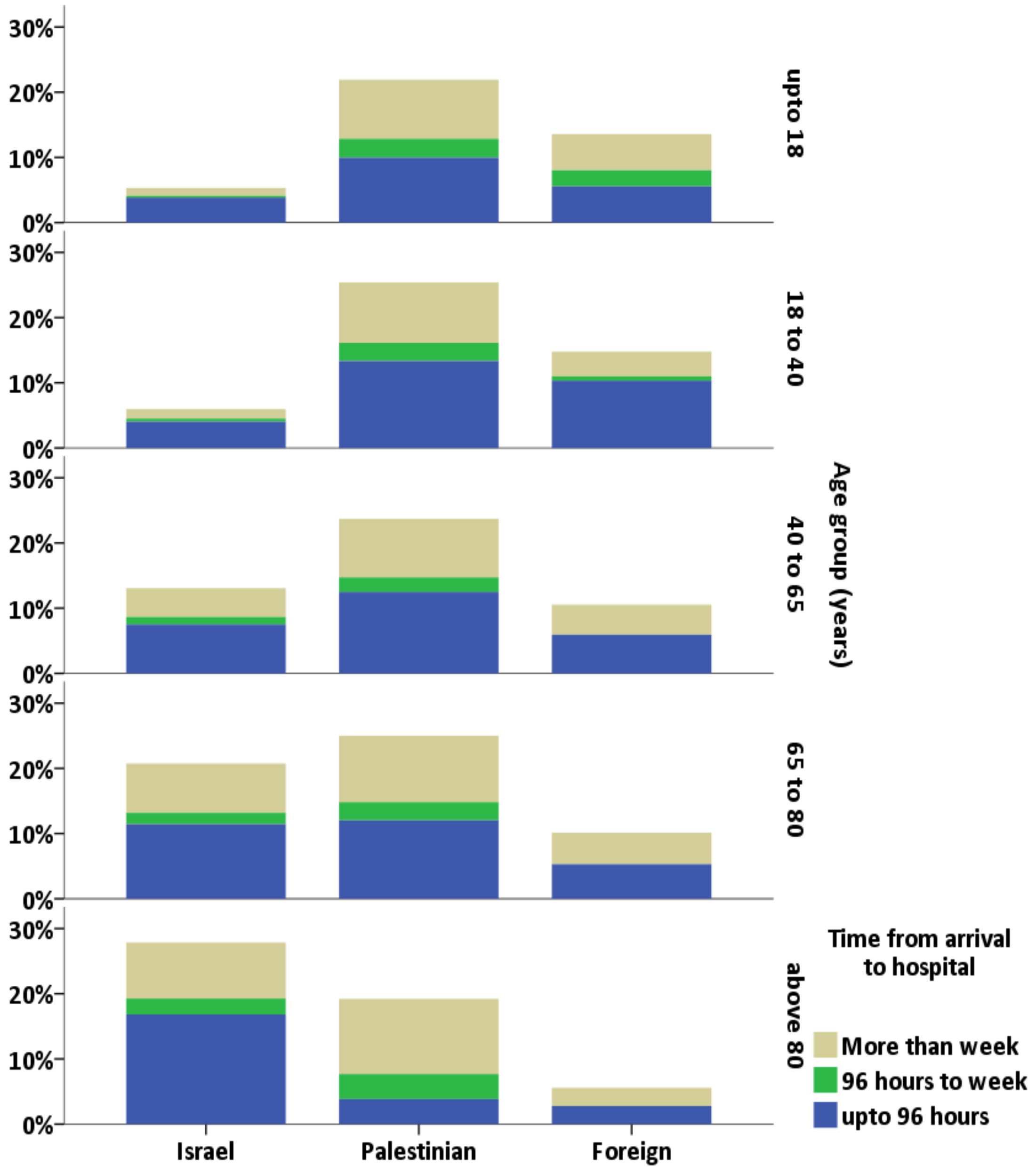

Multi drug resistant (MDR) prevalence per age group and source population, including extended-spectrum-beta-lactamase producing Enterobacteriaceae (ESBL-PE) and carbapenem-resistant *Pseudomonas aeruginosa* (CR-PA). Each panel depicts MDR prevalence per age group. Columns depict the cumulative MDR rate in each age group, per patient nationality. The color specifications depict the time from hospital admission to first identification of MDR bacteria.
